# Psychosocial factors promoting resilience during the menopausal transition

**DOI:** 10.1007/s00737-020-01055-7

**Published:** 2020-07-27

**Authors:** Hannah Süss, Jasmine Willi, Jessica Grub, Ulrike Ehlert

**Affiliations:** 1 Clinical Psychology and Psychotherapy, University of Zurich, Binzmühlestrasse 14, 8050 Zurich, Switzerland; 2 URPP Dynamics of Healthy Aging Research Priority Program, University of Zurich, Zurich, Switzerland

**Keywords:** Resilience, Perimenopause, Well-being, women’s mental health

## Abstract

Despite significant biological, psychological, and social challenges in the perimenopause, most women report an overall positive well-being and appear to be resilient to potentially negative effects of this life phase. The objective of this study was to detect psychosocial variables which contribute to resilience in a sample of perimenopausal women. A total of 135 healthy perimenopausal women aged 40–56 years completed a battery of validated psychosocial questionnaires including variables related to resilience, well-being, and mental health. First, using exploratory factor analysis, we examined which of the assessed variables related to resilience can be assigned to a common factor. Second, linear regression analyses were performed to investigate whether a common resilience factor predicts well-being and mental health in the examined sample of women. Optimism (LOT-R-O), emotional stability (BFI-K-N), emotion regulation (ERQ), self-compassion (SCS-D), and self-esteem (RSES) in perimenopausal women can be allocated to a single resilience-associated factor. Regression analyses revealed that this factor is related to higher life satisfaction (SWLS; *β* = .39, *p* < .001, adj. *R*^2^ = .20), lower perceived stress (PSS-10; *β* = − .55, *p* < .001, adj. *R*^2^ = .30), lower psychological distress (BSI-18; *β* = − .49, *p* < .001, adj. *R*^2^ = .22), better general psychological health (GHQ-12; *β* = − .49, *p* < .001, adj. *R*^2^ = .22), milder menopausal complaints (MRS II; *β* = − .41, *p* < .001, adj. *R*^2^ = .18), and lower depressive symptoms (ADS-L; *β* = − .32, *p* < .001, adj. *R*^2^ = .26). The *α* levels were adjusted for multiple testing. Our findings confirm that several psychosocial variables (optimism, emotional stability, emotion regulation, self-compassion, and self-esteem) can be allocated to one common resilience-associated factor. This resilience factor is strongly related to women’s well-being as well as mental health in perimenopause.

## Introduction

The perimenopause is associated with significant biological, psychological, and social changes and challenges (Jaspers et al. 2015; Greer 2018). As part of the natural decrease in ovarian hormone secretion, steroid hormone levels fluctuate in the perimenopause (Fiacco et al. 2019). Due to these hormonal changes, many women experience menopausal complaints such as hot flushes, night sweats, or sleep disturbances (Freeman et al. 2007). It is also a critical phase for the onset of psychological disorders such as depression and anxiety (Bromberger et al. 2007; Freeman 2010; Soares 2017). Besides this, changes and transitions in social relationships may occur during the perimenopause, such as children leaving home, the birth of grandchildren, dealing with an unfulfilled desire for children, marital tensions, or long-term care dependency or death of parents (Schmidt et al. 2004; Mishra and Kuh 2006).

As physical and psychological transitions and challenges may have an impact on the psychological well-being of perimenopausal women, specific coping strategies are required for a successful adjustment (Ngai 2019). Women who cope adequately with the challenges presented by the perimenopause can be described as resilient (Brown et al. 2015). Recently, we provided a conceptual framework to describe the complex interplay between symptoms of the perimenopause, coping with symptoms, resilience factors, and the resulting state on a continuum between psychological adjustment and maladjustment (Süss and Ehlert 2020). Previous work has already shown that high resilience is associated with milder menopausal symptoms as well as better psychological and physical well-being (Klohnen et al. 1996; Chedraui et al. 2012; Coronado et al. 2015).

However, previous definitions and measurements of resilience are not sufficient to capture all relevant aspects of resilience during this complex and highly specific psychophysiological transition phase in a woman’s life (Taylor-Swanson et al. 2018). Over the past decades, more than a dozen measurements have been developed to examine psychological resilience factors (Süss and Fischer 2019). Nevertheless, resilience researchers (Chmitorz et al. 2018) point out that there is no current ‘gold standard’ to assess resilience. Thus, due to the heterogeneity, a valid comparison between resilience measurement scales is not feasible at the present time (Süss and Ehlert, 2020).

Therefore, a broader range of resilience-associated variables should be considered. According to our framework, specific resilience factors are related to the perception of and the successful coping with perimenopausal changes. Current research encompasses various psychological concepts related to resilience, of which the most frequently used will be described here. Optimism seems to have a positive effect on the adaptation to menopausal symptoms (Caltabiano and Holzheimer 1999; Elavsky and McAuley 2009) and is associated with fewer depressive symptoms (Bromberger et al. 2015). Women with high emotional intelligence seem to experience less severe menopausal symptoms (Bauld and Brown 2009) and describe a higher health-related quality of life compared with women with lower emotional intelligence (Extremera and Fernández-Berrocal 2002; Bauld and Brown 2009). Emotional stability appears to explain a notable amount of variance in the adaptation to menopausal symptoms (Caltabiano and Holzheimer 1999) and is associated with less menopausal stress and fewer depressive symptoms (Bosworth et al. 2003; Mauas et al. 2014). Furthermore, high self-compassion and self-esteem negatively correlate with depressive symptoms (Brown et al. 2014; Mauas et al. 2014; Bromberger et al. 2015). While higher self-esteem appears to be a predictor of milder menopausal symptoms, higher self-compassion predicts better emotional balance (Brown et al. 2014). Both factors contribute to an increased quality of life and well-being. Taken together, some variables have already been examined with regard to their contribution to resilience during the menopausal transition. According to our resilience framework, the mentioned variables can be considered as resilience factors (Süss and Ehlert 2020). The present study represents an empirical validation of this resilience concept.

The primary aim of the study was to detect psychosocial variables which contribute to resilience in perimenopausal women. For this purpose, we examined which of the assessed variables related to resilience can be assigned to a common factor. Up to now, this has never been investigated in the context of perimenopause. The clarification and assignment of essential psychosocial variables contributing to resilience in perimenopausal women will help us to achieve a more precise operationalization of this construct. The second objective was to investigate the relation of resilience with different aspects of health and well-being to empirically validate this resilience concept. Achieving these two goals, in turn, enables us to identify potential starting points to foster psychological well-being and health in perimenopausal women.

## Materials and methods

This study is part of the Swiss Perimenopause Study, a large research project that is currently being conducted by our workgroup at the Institute of Psychology, University of Zurich, Switzerland. The Cantonal Ethics Committee of the canton of Zurich and the Cantonal Data Protection Commission of the canton of Zurich approved the study (KEK-ZH-Nr. 2018-00555). The study was conducted in accordance with the principles of the Declaration of Helsinki. One central aim of this research project is to gain a deeper understanding of the biopsychosocial factors and processes associated with resilience and health in the perimenopause.

In the Swiss Perimenopause Study, a large variety of psychosocial variables are evaluated in a sample of perimenopausal women. The present study is a cross-sectional investigation of the sample at assessment time point 1, 3 months after enrolment. A detailed description of the study protocol can be found elsewhere (Willi et al. 2020).

### Participants and procedure

In total, data from 135 self-reporting healthy perimenopausal 137 women aged 40–56 years were assessed (see also Willi et al. 2020). All women were recruited through social media, mailing lists, women’s health-related online forums, and flyers as well as newspaper and magazine articles. At enrollment, participants had to report either a good, very good, or excellent health condition, and had to state that they were currently free of any acute or chronic somatic disease or mental disorder. To ensure that all participants were mentally healthy at the beginning of the study, a well-instructed psychologist conducted the German version of the Structured Clinical Interview for DSM-IV (Wittchen et al., 1997). The perimenopausal status was assessed with the Stages of Reproductive Aging Workshop *+10* (STRAW) criteria (Harlow et al. 2012). Of 1121 women interested in participating in the Swiss Perimenopause Study, a total of 986 had to be excluded, mainly due to pre- or postmenopausal status (*n* = 717), poor subjective health (*n* = 97), age below 40 or above 60 years (*n* = 58), hormone therapy in the last 6 months (*n* = 34), acute or chronic somatic or mental illness (*n* = 22), and psychiatric or psychotropic drug use (*n* = 11). For detailed information, see (Willi et al., 2020). Reported data were collected between August 2018 and January 2020. All measures used in this study were assessed via online self-reports using validated questionnaires. Prior to completing the questionnaires, participants were explicitly informed about the procedure and the expected duration (30 min per package) of the online assessment. Participants were required to complete the four questionnaire packages consecutively.

### Variables related to resilience

Current research encompasses various psychosocial concepts related to resilience in perimenopausal women (Süss and Ehlert 2020). In the present study, the most frequently used traits were examined using the following validated questionnaires:

*Optimism* seems to influence the shaping of cognitive schemata, leading to differences in the awareness and reporting of menopausal symptoms (Elavsky and McAuley 2009). Dispositional optimism was assessed using the German version of the Life Orientation Test-Revised (LOT-R; Glaesmer et al., 2008). The ten items (three items each for optimism and pessimism, four neutral items) are summed up to form an optimism and a pessimism scale. Only the optimism scale (LOT-R-O) was used in this study.

*Emotional stability* appeared to explain a reasonable amount of variance in adaptation to perceived menopausal stress (Bosworth et al. 2003) and symptoms (Caltabiano and Holzheimer 1999). It was measured with the German short version of the Big Five Inventory (BFI-K; Rammstedt & John, 2005). The BFI-K includes the Big Five personality traits: extraversion, agreeableness, conscientiousness, neuroticism (vs. emotional stability), and openness to experience. In this study, only the neuroticism subscale (BFI-K-N) was used, as low neuroticism scores represent high emotional stability (Hill et al. 2013).

A better *emotion regulation* was found to predict lower levels of depressive symptoms during the menopausal transition (Mauas et al., 2014). The validated German version of the Emotion Regulation Questionnaire (ERQ; Abler & Kessler, 2009) was used to assess two common trait emotion regulation strategies, i.e., reappraisal and suppression. According to Goleman (1995), emotion regulation is one of five facets of emotional intelligence.

*Self-compassion* includes having an understanding and a nonjudgmental attitude toward oneself (Neff 2003). Since it decreases over the course of life, and the perimenopause is considered a high-risk life event in this regard (Deeks and McCabe 2004), it plays a key role in perceiving low stress levels during this phase of life (Abdelrahman et al. 2014). Participants’ self-compassion was measured with the validated short-form German translation of the Self-Compassion Scale (SCS-D; Hupfeld & Ruffieux, 2011).

Previous studies have already shown that women with a higher *self-esteem* adapt better to perceived stress (Guérin et al., 2017), show lower levels of depressive symptoms (Guérin et al., 2017), and report fewer menopausal complaints (Koch et al., 2017; Zhang et al., 2016). As one of the most widely used instruments in this particular area, the German version of the Rosenberg Self-Esteem Scale (RSES; von Collani and Herzberg 2003) was used to examine participants’ self-esteem. The higher the total score, the higher the level of self-esteem.

### Assessment of well-being and mental health

Based on our recent review paper (Süss and Ehlert, 2020), we claim that higher resilience in perimenopausal women is associated with a better adjustment to this life phase. Across the reviewed studies, this adjustment was operationalized by higher satisfaction with life, less perceived stress, better psychological well-being, a better adjustment to menopausal symptoms, or fewer depressive symptoms compared with women with lower resilience. Thus, the assessment of well-being and mental health included the following validated questionnaires:

*Life satisfaction* was assessed using the German version of the Satisfaction with Life Scale (SWLS; Glaesmer et al., 2011). The SWLS is the most commonly used instrument in this area.

Participants completed the validated German translation of the Perceived Stress Scale (PSS-10; Klein et al., 2016), a 10-item self-report questionnaire that quantifies a participant’s subjective *stress experience* within the last month.

Participants’ *psychological distress* was examined using the validated German translation of the Brief Symptom Inventory (BSI-18; Spitzer et al., 2011). The sum score represents a measure of general psychological distress including somatization, depression, and anxiety.

The validated German version of the 12-item General Health Questionnaire (GHQ-12; Schmitz et al., 1999) was used to evaluate *general psychological health*. The items sum up to a possible score ranging from 0 to 36, with higher scores representing higher levels of distress.

The subjective assessment of *menopausal complaints* was measured using the Menopause Rating Scale (MRS II; Hauser et al., 1999). Women rated the occurrence and severity of menopausal symptoms on eleven items, which were summed up to form a total score.

The *Allgemeine Depressionsskala* (ADS-L; Hautzinger & Bailer, 1993) is the revised German translation of the Center for Epidemiological Studies Depression Scale (CES-D; Radloff, 1977). The CES-D is by far the most frequently used questionnaire to assess depressive symptoms in menopausal women (Willi and Ehlert 2019).

### Data analysis

All statistical analyses were conducted using the Statistical Package for the Social Sciences (SPSS, version 25). For the sample size estimation, we performed an a priori sample size estimation analysis using GPower 3.1 (Faul et al. 2009). Prior to statistical analysis, a descriptive evaluation of the collected data was carried out.

Subsequently, we examined which of the assessed variables related to resilience can be reduced to a common factor using exploratory factor analysis. Therefore, we first calculated the correlation coefficients (correlation matrix) of these variables (Ledesma et al. 2015). Prior to the extraction of the factors, we assessed the suitability of the data for factor analysis by conducting the Kaiser-Meyer-Olkin (KMO) test and Bartlett’s test of sphericity (Dziuban and Shirkey 1974). Furthermore, the Kaiser criterion (eigenvalue > 1; Kaiser, 1961) and Cattell’s scree test (Cattell 1966) were examined to determine the number of factors (Ledesma et al. 2015). After resolving the number of factors and their extraction, we conducted a principal component analysis to assess the size of factor loadings (correlations between the variables being tested and the factor) (Tinsley and Tinsley 1987).

Simple linear regression analyses were performed with the resilience-associated factor as the independent variable and different aspects of well-being and mental health as the dependent variable. Prior to statistical analyses, assumptions of linear regression were tested (Casson and Farmer 2014). For all analyses investigating the association of the resilience-associated factor with women’s well-being and mental health, we included age, BMI, annual household income, and menopausal status (early or late perimenopause) as covariates. Additionally, history of depression was statistically considered when testing the association of the resilience-associated factor and depressive symptoms (ADS-L; Hautzinger & Bailer, 1993). Prior depression must have either been self-reported, in accordance with the criteria for major depression according to the Diagnostic and Statistical Manual of Mental Disorders, Fifth Edition (DSM-5), or previously diagnosed by an expert. The false discovery rate was applied to the *α* level (normally *p* < .05) to control the overall type 1 error rate taking into account the number of six significance tests (Benjamini and Hochberg 1995). Missing data from participants were excluded listwise from subsequent analyses. Subjects scoring below − 28 (*n* = 5) on the social desirability scale were excluded from analyses including the ADS-L (Hautzinger and Bailer 1993).

## Results

### Sample characteristics

Table 1 provides sociodemographic and health-related sample characteristics. Most of the participants were Swiss (85.2%), married (56.3%), well-educated, and of normal weight (*M* = 23.38 kg/m^2^, *SD* = 3.69).

**Table 1:** Descriptive statistics of sociodemographic and health-related sample characteristics (*N* = 135)

	*N* (%)	*M* (*SD*)
Age		48.60 (3.87)
Nationality		
Swiss	115 (85.2%)	
German	17 (12.6%)	
Other	3 (2.1%)	
Marital status		
Single	34 (25.2%)	
Married	76 (56.3%)	
Registered partnership	4 (3.0%)	
Divorced	18 (13.3%)	
Widowed	3 (2.2%)	
Children		
Yes	91 (67.4%)	
No	44 (32.6%)	
Highest educational attainment		
Secondary school	50 (37.0%)	
Grammar school	27 (20.0%)	
University	58 (43.0%)	
Occupational situation		
Homemaker	6 (4.4%)	
Employee	92 (68.2%)	
Manager	37 (27.4%)	
Annual household income (CHF)		165,092 (174,815)
Menopausal stage		
Early MT	59 (43.7%)	
Late MT	76 (56.3%)	
History of depression		
No	81 (60.0%)	
Yes	54 (40.0%)	
BMI		23.38 (3.69)

*N* sample size, *M* mean, *SD* standard deviation, *CHF* Swiss Francs, *MT* menopausal transition, *BMI* body mass index

Descriptive statistics of variables related to resilience as well as well-being and mental health are presented in Table 2.

**Table 2:** Descriptive statistics of variables related to resilience as well as well-being and mental health

	*N*	*M ± SD*	MIN, MAX	Range
Variables related to resilience				
LOT-R-O	134	9.00 ± 2.43	1.00, 12.00	0–12
BFI-K-N	134	11.43 ± 3.04	4.00, 17.00	4–20
ERQ	130	4.09 ± 0.92	1.30, 6.30	1–7
SCS-D	134	3.45 ± 0.61	1.42, 4.83	1–5
RSES	134	32.51 ± 4.19	21.00, 40.00	10–40
Well-being and mental health				
SWLS	128	27.81 ± 4.27	10.00, 34.00	5–35
PSS-10	128	13.50 ± 5.14	4.00, 29.00	0–40
BSI-18	129	7.97 ± 6.49	0.00, 40.00	0–72
GHQ-10	129	11.42 ± 5.22	4.00, 29.00	0–36
MRS II	135	10.36 ± 5.49	1.00, 29.00	0–44
ADS-L	130	12.58 ± 9.10	1.00, 29.00	0–60

Data are presented as mean (*M*) ± standard deviation (*SD*); minimum (*MIN*), maximum (*MAX*) scores; and range. Except for emotional stability (BFI-K-N) and general psychological health (GHQ-10), higher scores indicate higher levels of the respective domains

### Factor analysis

The primary aim of this study was to clarify which of the assessed psychosocial variables related to resilience can be assigned to a common factor using an exploratory factor analysis.

We first calculated the correlation coefficients of optimism, emotional stability, emotion regulation, self-compassion, and self-esteem, displaying the relationships between individual variables (Ledesma et al. 2015). Table 3 shows the Pearson correlation matrix.

**Table 3:** Correlation table of variables related to resilience

	LOT-R-O	BFI-K-N	ERQ	SCS-D	RSES
LOT-R-O	1.00	− .45***	.18*	.43***	.39***
BFI-K-N		1.00	− .22**	− .54***	− .49***
ERQ			1.00	.17*	.08
SCS-D				1.00	.65***
RSES					1.00

Significance level (one-tailed): *p < .05, **p < 0.01, ***p < 0.001

The KMO test verified the sampling adequacy for the analysis with a score of .77, and Bartlett’s test was significant (*χ*^2^ (10) = 165.10, *p* < .001). Therefore, it can be assumed that our data are suitable for factor analysis (Dziuban and Shirkey 1974).

**Fig. 1:**
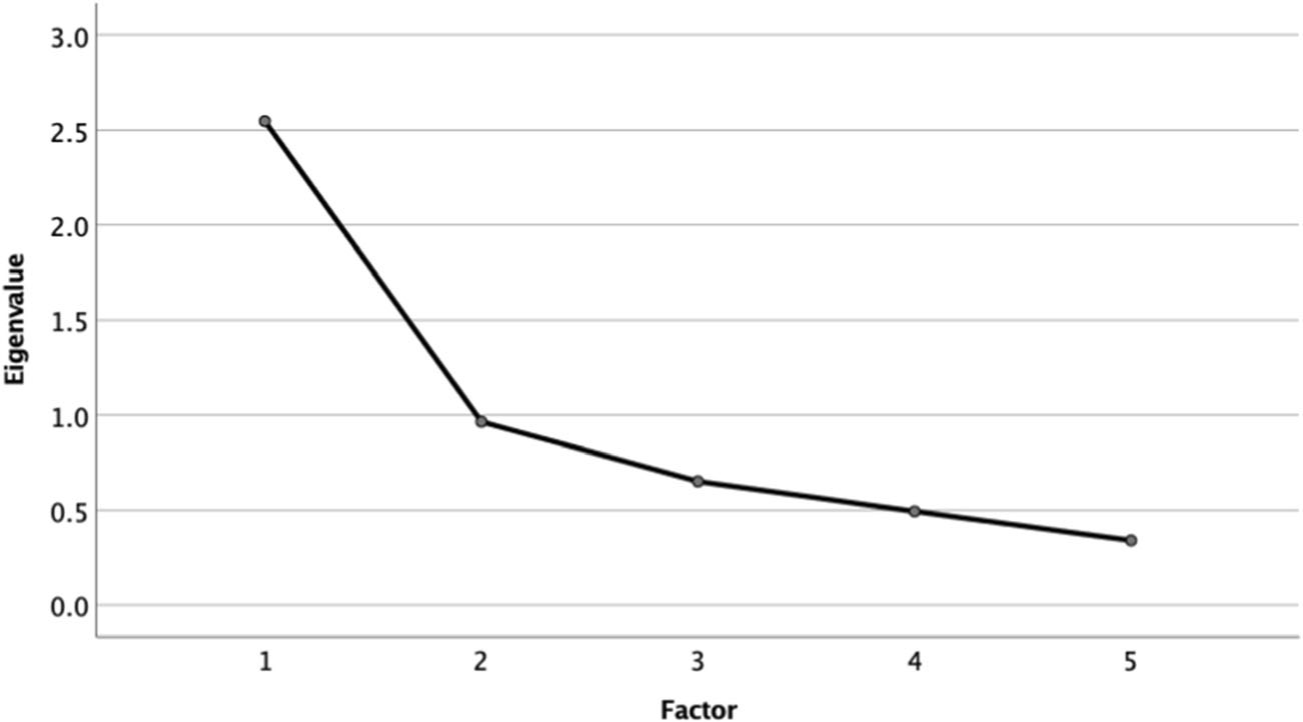
Scree plot

According to the Kaiser criterion (Kaiser 1961), one factor (explaining *51.00%* of the variance) can be extracted. Based on the scree test (Cattell 1966), the relevant factors are those whose eigenvalues lie in front of the sharp bend in the scree plot (Fig. 1). Therefore, both the Kaiser criterion and scree plot indicate that a single factor should be retained.

Independent of the sample size, a factor can be interpreted if at least four variables have a loading of ± .60 or more. Additional factor loadings above ± .30, which equates to approximately 10% overlapping variance with the other sum scores, should be considered in the interpretation of a factor (Tinsley and Tinsley 1987). Table 4 shows that optimism, emotional stability, self-compassion, and self-esteem have high loadings on the extracted factor, whereas emotion regulation has lower but acceptable loadings on the one extracted factor solution.

**Table 4:** Factor loadings of variables related to resilience

Variable	Factor
	1
Self-compassion (SCS-D)	.835
Self-esteem (RSES)	.794
Emotional stability (BFI-K-N)	− .793
Optimism (LOT-R-O)	.698
Emotion regulation (ERQ)	.325

*SCS-D*, Self-Compassion Scale; *RSES*, Rosenberg Self-Esteem Scale; *BFI-K*, Short version of the Big Five Inventory; *LOT-R-O*, Life Orientation Test-Revised Optimism; *ERQ*, Emotion Regulation Questionnaire

Finally, participants in the early and late perimenopausal stage were investigated independently by running two further factor analyses. In both cases, the Kaiser criterion and scree plot indicated that a single factor should be retained. Thus, there seems to be no difference regarding psychosocial variables contributing to resilience in these two groups.

### Associations between the resilience-associated factor and well-being and mental health

**Fig. 2:**
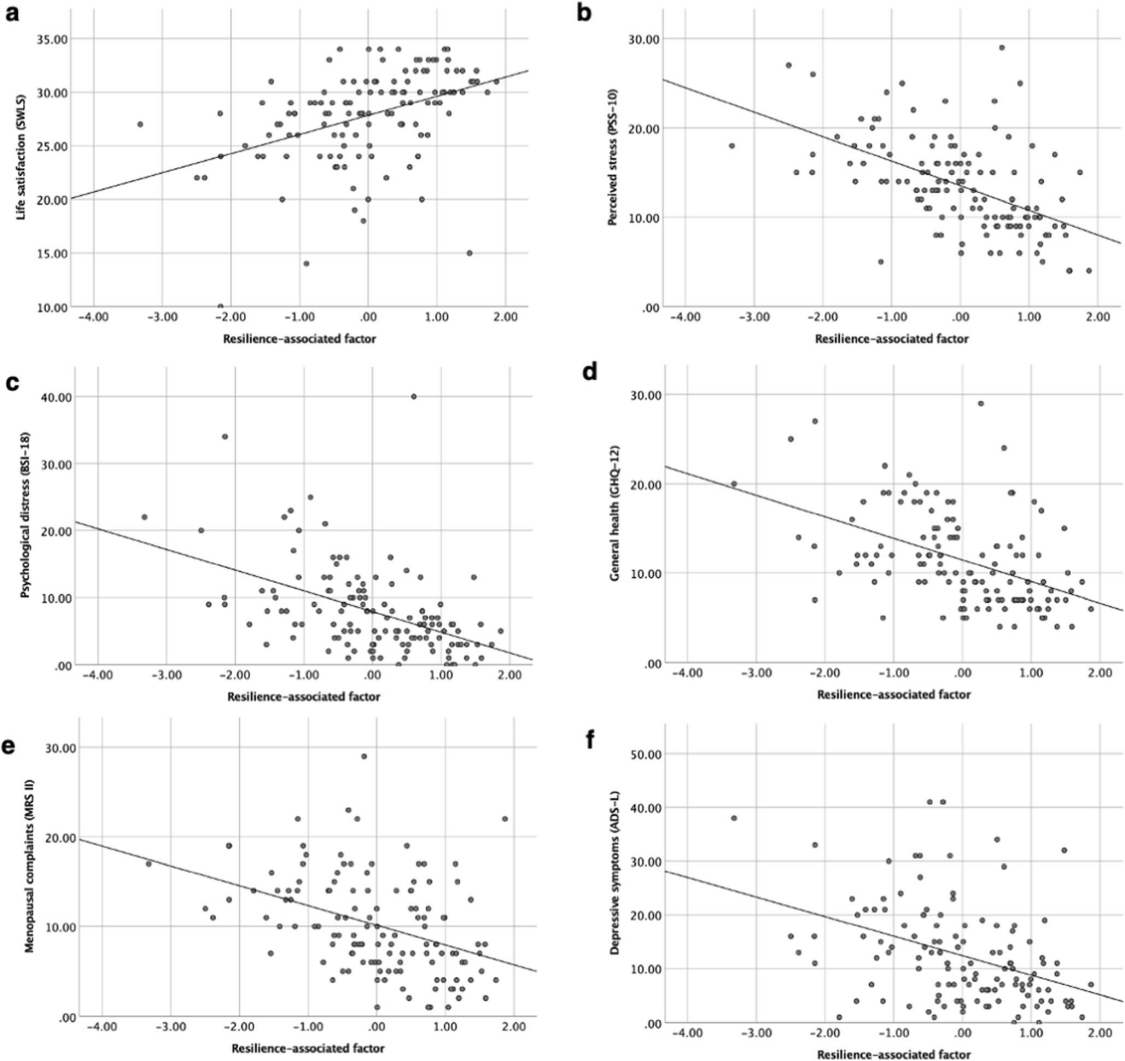
Scatter plots of the linear associations between the extracted resilience-associated factor and different aspects concerning well-being and mental health. Higher scores on this resilience-associated factor are significantly related to **a** higher life satisfaction (SWLS), **b** lower perceived stress (PSS-10), **c** lower psychological distress (BSI), **d** better general psychological health (GHQ-12), **e** milder menopausal complaints (MRS II), and **f** lower depressive symptoms (ADS-L). Except for general psychological health (GHQ-10), higher scores indicate higher levels of the respective domains

As a second aim, we investigated the relation of the resilience-associated factor with a variety of aspects of well-being and mental health. For this purpose, simple linear regression analyses were conducted. All findings were statistically adjusted for multiple testing (Benjamini and Hochberg 1995). Regression analyses revealed that higher scores on this resilience-associated factor are associated with higher life satisfaction (SWLS; *β* = .39, *p* < .001, adj. *R*^2^ = .20), lower perceived stress (PSS-10; *β* = − .55, *p* < .001, adj. *R*^2^ = .30), lower psychological distress (BSI-18; *β* = − .49, *p* < .001, adj. *R*^2^ = .22), better general psychological health (GHQ-12; *β* = − .49, *p* < .001, adj. *R*^2^ = .22), milder menopausal complaints (MRS II; *β* = − .41, *p* < .001, adj. *R*^2^ = .18), and lower depressive symptoms (ADS-L; *β* = − .32, *p* < .001, adj. *R*^2^ = .26). Scatter plots visualizing these linear associations between the extracted resilience-associated factor and different aspects of well-being and mental health can be found in Fig. 2. All results remained significant after additionally controlling for history of depression in all analyses.

## Discussion

The primary objective of the present analyses was to detect which psychosocial variables contribute to resilience in perimenopausal women. The results showed that optimism, emotional stability, emotion regulation, self-compassion, and self-esteem can be assigned to one resilience-associated factor. As a second aim, we investigated the relation of resilience with a variety of aspects of well-being and mental health. Our findings indicate that women with higher resilience seem to have better well-being and report better mental health in the perimenopause.

**Fig. 3:**
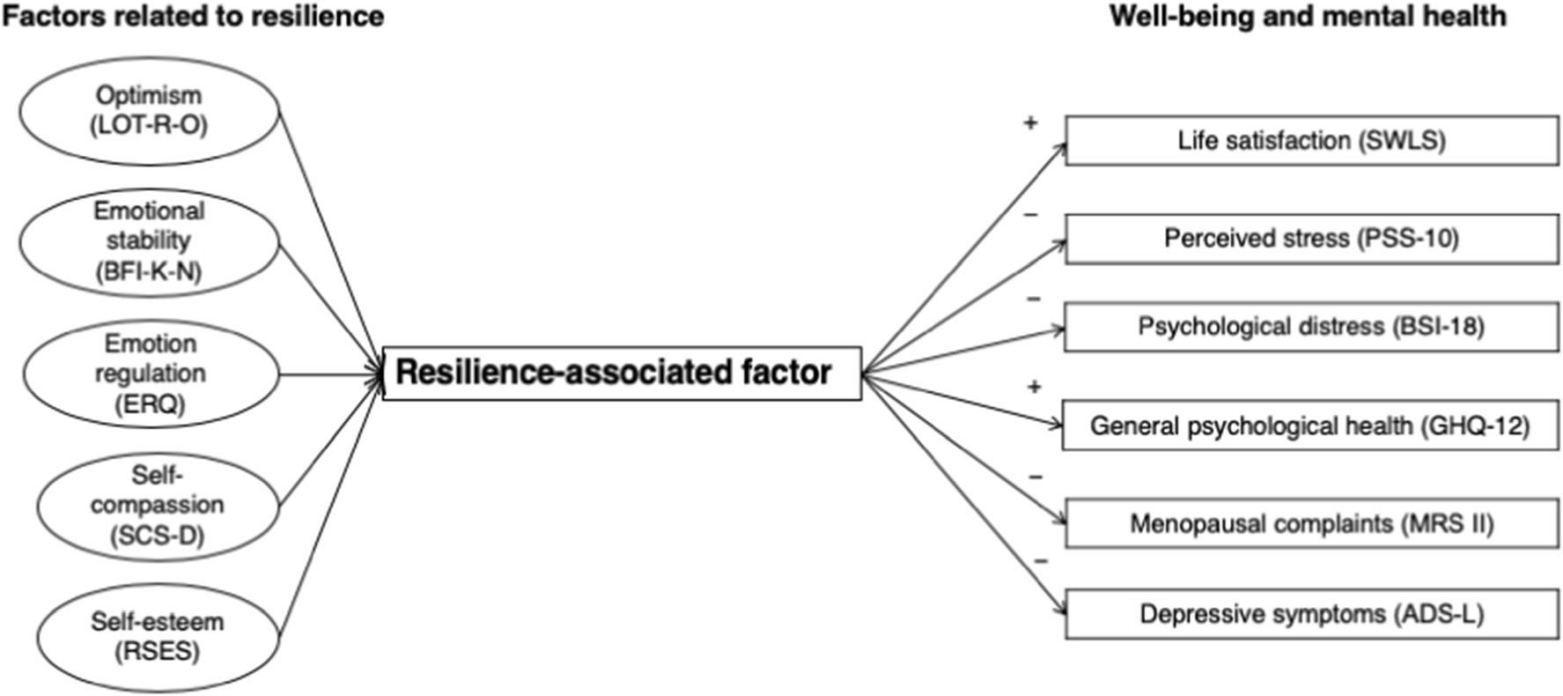
Psychosocial factors promoting resilience in perimenopausal women. LOT-R-O, Life Orientation Test-Revised Optimism; BFI-K, short version of the Big Five Inventory; ERQ, Emotion Regulation Questionnaire; SCS-D, Self-Compassion Scale; RSES, Rosenberg Self-Esteem Scale; SWLS, Satisfaction with Life Scale; PSS-10, Perceived Stress Scale; BSI-18, Brief Symptom Inventory; GHQ-12, General Health Questionnaire; MRS II, Menopause Rating Scale; ADS-L, Allgemeine Depressionsskala

This study represents an empirical validation of our previously introduced framework of resilience as defined by the novel resilience-associated factor (Fig. 3). Several authors have examined these resilience variables individually with regard to their contribution to resilience during the menopausal transition (Caltabiano and Holzheimer 1999; Extremera and Fernández-Berrocal 2002; Bosworth et al. 2003; Bauld and Brown 2009; Elavsky and McAuley 2009; Brown et al. 2014). Since the resilience-associated variables investigated across previous studies are very heterogeneous (Süss and Ehlert 2020), the present study considered a broad range of resilience-associated variables within the same sample of perimenopausal women. Furthermore, previous definitions and measurements of resilience are not sufficient to capture the broader aspects of resilience during this phase of transition in a woman’s life. Therefore, the clarification and assignment of essential psychosocial variables contributing to resilience in perimenopausal women helped us to achieve a more precise operationalization of this construct.

Accordingly, we were able to present exciting research findings by confirming the associations between resilience and a comprehensive range of factors of health and well-being within the same sample of perimenopausal women. Previous studies only investigated selected associations between resilience variables and resulting states of adjustment or maladjustment (e.g., Bauld & Brown, 2009; Brown et al., 2014; Caltabiano & Holzheimer, 1999; Elavsky & McAuley, 2009). The present study confirmed the associations between high resilience and high life satisfaction, low perceived stress, low psychological distress, good general psychological health, mild menopausal complaints, and low depressive symptoms within the same sample of perimenopausal women. Thus, our findings suggest the need to consider resilience-associated variables like optimism, emotional stability, emotion regulation, self-compassion, and self-esteem as potential starting points to foster psychological well-being and health during perimenopause. Since resilience is a dynamic process (Chmitorz et al. 2018), the results of the present studies could be used to further develop early interventions (Hunter and Mann 2010) in order to enhance resilience and increase women’s well-being and mental health in this life phase.

The presented empirical validation of a resilience concept in perimenopausal women is highly relevant. During and even after perimenopause, resilience plays a central role in facing the multifaceted challenges inherent in the aging process, being associated with self-rated successful aging, with an effect comparable in size with that for physical health (Jeste et al. 2013). While selected studies have investigated the concept of psychological resilience in middle and older age (Lamond et al. 2008; Resnick and Inguito 2011; Gooding et al. 2012; Martin et al. 2015), the present study is the first to validate a comprehensive resilience concept focusing on perimenopausal women only. This is of crucial importance, since perimenopausal women not only face the general challenges of aging but also are at the same time confronted with the specific biopsychosocial changes and complaints of this transition phase.

The major strength of our study is the examination of healthy women in the perimenopause only, through the deployment of strict inclusion criteria. Previous studies frequently included women across different stages of reproductive aging (pre-, peri-, and postmenopause), whereas the present study investigated only perimenopausal women on the basis of bleeding patterns using the gold standard STRAW criteria (Harlow et al. 2012). In this respect, a distinction between early and late perimenopausal women was taken into account as a covariate. A specific focus on the perimenopause, which is an especially critical window of increased biopsychosocial changes and associated complaints, seems to be essential in order to specify the relations between resilience and health and well-being in this life phase. By investigating a physically and mentally healthy population of perimenopausal women, we were able to keep confounders from interventions and treatments to a minimum. Besides this, participants were randomly identified from the general population and therefore represent an eligible sample for this study. Moreover, participants completed a wide range of validated psychosocial questionnaires, helping to provide a very comprehensive picture of women’s resilience, well-being and health in perimenopause. To date, only a small number of comprehensive resilience concepts have been empirically validated, primarily referring to resilience in stress-associated professions (Gillespie et al. 2007; Skomorovsky and Stevens 2013; De Terte et al. 2014) and chronic diseases (Vinson 2002; Haase 2004). The present study contributed to further achieving a more precise operationalization of this construct.

Nevertheless, there are some limitations which need to be considered when interpreting the findings of our study. First, the present study is a cross-sectional investigation. Since data were gathered at only one time point, it was not possible to infer causality of the tested associations. Longitudinal data will clarify whether our extracted resilience-associated factor has predictive power. Second, by assessing emotion regulation (ERQ; Abler & Kessler, 2009), we covered only one of five facets of emotional intelligence. Future work should incorporate all of the facets proposed by Goleman (1995) in order to confirm not only emotion regulation but also emotional intelligence as a resilience-associated factor. Third, all assessed psychosocial measures were self-reported, which might generate a response bias. To reduce the likelihood of socially desirable responding, questionnaires were completed online and encrypted with a personal code. However, future research may also consider objective health outcome measures such as physiological and biological parameters. Finally, considering that the perimenopause is a bio-psycho-social process (Hunter and Rendall 2007), future research should also include the assessment of biological markers of resilience. Some authors have already identified biological underpinnings of resilience (Charney 2004; Russo et al. 2012; Feder et al. 2013). However, there are no studies assessing such correlates for the perimenopause.

## Conclusions

The present study delivers an empirical validation of the theoretical concept of resilience by detecting that optimism, emotional stability, emotion regulation, self-compassion, and self-esteem can be assigned to one common resilience-associated factor. The results reveal that psychosocial resilience is linked to women’s psychological well-being and health during perimenopause. The present findings suggest the need to consider resilience variables as potential starting points from which to foster higher life satisfaction, lower stress, improve mental health, and achieve milder menopausal complaints in perimenopausal women.

## Abbreviations

ADS-L: Allgemeine Depressionsskala
BFI-K: short version of the Big Five Inventory
BMI: body mass index
BSI-18: Brief Symptom Inventory
CES-D: Center for Epidemiological Studies Depression Scale
CHF: Swiss Francs
DSM-5: Diagnostic and Statistical Manual of Mental Disorders, Fifth Edition
ERQ: Emotion Regulation Questionnaire
GHQ-12: General Health Questionnaire
KMO: Kaiser-Meyer-Olkin
LOT-R-O: Life Orientation Test-Revised Optimism
MRS II: Menopause Rating Scale
PSS-10: Perceived Stress Scale
RSES: Rosenberg Self-Esteem Scale
SCID: Structured Clinical Interview for DSM-IV
SCS-D: Self-Compassion Scale
SPSS: Statistical Package for the Social Sciences
STRAW: Stages of Reproductive Aging Workshop
SWLS: Satisfaction with Life Scale
